# Root Transcriptome and Metabolome Profiling Reveal Key Phytohormone-Related Genes and Pathways Involved Clubroot Resistance in *Brassica rapa* L.

**DOI:** 10.3389/fpls.2021.759623

**Published:** 2021-12-15

**Authors:** Xiaochun Wei, Yingying Zhang, Yanyan Zhao, Zhengqing Xie, Mohammad Rashed Hossain, Shuangjuan Yang, Gongyao Shi, Yanyan Lv, Zhiyong Wang, Baoming Tian, Henan Su, Fang Wei, Xiaowei Zhang, Yuxiang Yuan

**Affiliations:** ^1^Institute of Horticulture, Henan Academy of Agricultural Sciences, Graduate T&R Base of Zhengzhou University, Zhengzhou, China; ^2^Henan International Joint Laboratory of Crop Gene Resources and Improvement, School of Agricultural Sciences, Zhengzhou University, Zhengzhou, China; ^3^Department of Genetics and Plant Breeding, Bangladesh Agricultural University, Mymensingh, Bangladesh

**Keywords:** clubroot resistance, RNA-seq, phytohormone, metabolome profiling, *Brassica rapa* L., *P. brassicae*

## Abstract

*Plasmodiophora brassicae*, an obligate biotrophic pathogen-causing clubroot disease, can seriously affect *Brassica* crops worldwide, especially Chinese cabbage. Understanding the transcriptome and metabolome profiling changes during the infection of *P. brassicae* will provide key insights in understanding the defense mechanism in *Brassica* crops. In this study, we estimated the phytohormones using targeted metabolome assays and transcriptomic changes using RNA sequencing (RNA-seq) in the roots of resistant (BrT24) and susceptible (Y510-9) plants at 0, 3, 9, and 20 days after inoculation (DAI) with *P. brassicae*. Differentially expressed genes (DEGs) in resistant vs. susceptible lines across different time points were identified. The weighted gene co-expression network analysis of the DEGs revealed six pathways including “Plant–pathogen interaction” and “Plant hormone signal transduction” and 15 hub genes including pathogenic type III effector avirulence factor gene (*RIN4*) and auxin-responsive protein (*IAA16*) to be involved in plants immune response. Inhibition of Indoleacetic acid, cytokinin, jasmonate acid, and salicylic acid contents and changes in related gene expression in R-line may play important roles in regulation of clubroot resistance (CR). Based on the combined metabolome profiling and hormone-related transcriptomic responses, we propose a general model of hormone-mediated defense mechanism. This study definitely enhances our current understanding and paves the way for improving CR in *Brassica rapa*.

## Introduction

Clubroot is a soil-borne disease caused by *Plasmodiophora brassicae*, an obligate biotrophic protist that specifically infects cultivated and wild species of Brassicaceae family, including Chinese cabbage, cabbage, radish, cauliflower, and mustard, etc. (Howard et al., 2010). During infection, *P. brassicae* initially infects root hairs followed by the root cortex leading to root swelling and formation of gall. These galls hinder the uptake of water and nutrients, and result in abnormal shoot growth (Kageyama and Asano, 2009). The dormant spores of *P. brassicae* are highly persistent and may remain infectious in the soil for up to 20 years (Dixon, 2009; Kageyama and Asano, 2009). This disease causes serious damage to crop quality and yield resulting in a global yield loss of 10–15% in Brassicaceae plants (Dixon, 2009). In recent years, the incidence of clubroot disease is gradually increasing in China, with the southwest, northeast, and central regions being seriously affected (Zhu et al., 2019).

The initial defense of the plant against pathogens is manifested by physical (such as cell wall, cuticle, waxy layer, and xylogen, etc.) or chemical barriers [such as phenols, saponins, and glucosinolates (GSLs), etc.]. Once the above defense is breached, plants activate pathogen-associated molecular pattern (PAMP)-triggered immunity (PTI) system immediately. PTI consists of recognition and inhibition of PAMPs by pattern recognition receptors (PRRs) localized on the plasma membrane (Jones and Dangl, 2006). Pathogens can suppress PTI by secreting effectors into host cells leading to the activation of effector-triggered immunity (ETI). ETI is mediated by intracellular receptors encoded by plant disease resistance genes (R genes) that recognize the effector proteins and subsequently activate downstream immune responses to prevent pathogen infection (Sagi et al., 2017). Profiling the transcriptional changes and hormone signaling during these PTI and ETI is crucial for understanding the defense responses of plants against the pathogen (Moore et al., 2011).

In recent years, several “-omics” studies have focused on understanding the molecular mechanisms of clubroot resistance (CR) (Jia et al., 2017; Fu et al., 2019; Ning et al., 2019; Summanwar et al., 2021; Yuan et al., 2021). In *Brassica*, several studies indicated that the genes involved in cell-wall modification, SA signal transduction, phytoalexin synthesis, chitinase synthesis, Ca^2+^ signaling, reactive oxygen gene activation, the signaling metabolism of jasmonate and ethylene (ET), defensive deposition of callose, and the biosynthesis of indole-containing compounds were all significantly upregulated in clubroot-resistant plants (Devos et al., 2006; Chu et al., 2014; Zhang et al., 2016; Li et al., 2019; Zhou et al., 2020). A recent research showed that phenylpropanoid pathway was instrumental in resistance to clubroot disease progression in resistant line (Irani et al., 2019).

The mechanism of stress response in plants is highly intricate and requires several integrated pathways to be activated in response to external stresses. Because of the complex interactions among various plant hormones and their ability to control a wide range of physiological processes, they serve as the key endogenous factors in mediating plant stress response. Schuller et al. (2014) studied the role of auxin, cytokinin (CTK), and brassinosteroid (BR) signaling and metabolism in the clubroot development based on transcriptomic analysis and laser microdissection of *Arabidopsis* roots. Signaling and metabolic activity of jasmonate acid (JA) and ET were significantly upregulated in resistant lines compared to the susceptible lines at 15 days post-inoculation, whereas no increase in the expression of the genes involved in salicylic acid (SA) metabolism and signaling pathways were detected (Chu et al., 2014). Active CTKs and auxin were found to be elevated in both leaves and roots of infected plants, and applications of SA and JA were found to diminish and promote gall formation, respectively, in *Brassica napus* (Prerostova et al., 2018). It was found that abscisic acid (ABA) and ABA response-related genes, *RAB18*, *RD20*, and *RD22* accumulate in response to dehydration which may be involved in wilting of leaves in the later stages of the disease (Devos et al., 2005; Siemens et al., 2006).

In *Brassica rapa*, the transcriptomic and metabolomic changes during clubroot disease are not yet to be completely understood. Here, we study the changes in the metabolome profiling and hormone-related transcriptomic at different stages of clubroot development in the contrastingly resistant *B. rapa* genotypes. Our results provide new insights into the root transcriptome- and phytohormone-mediated defense responses at different stages of clubroot disease in *B. rapa*.

## Materials and Methods

### Plant Materials and *Plasmodiophora brassicae* Inoculum

The two *B. rapa* accessions, BrT24 (resistant, R-line) and Y510-9 (susceptible, S-line) having contrasting resistance to *P. brassicae* were obtained from the Institute of Horticulture, Henan Academy of Agricultural Sciences, China. The *P. brassicae* strain used in this study was collected from clubroot of *B. rapa* in Xinye County, Henan Province, China and identified as race 4 by the Williams system (Yuan et al., 2017). The *P. brassicae* spore suspension, diluted to 1 × 10^7^/ml was used as inoculum (Luo et al., 2014).

The seeds of R- and S-lines were germinated on wet filter paper followed by transplanting into 50-well plastic trays and incubating at a temperature of 25°C/20°C and photoperiod of 16 h/8 h (light/dark). After 20 days, each well was inoculated with 20 ml of *P. brassicae* solution with the control group receiving 20 ml of sterile water. Root samples were collected on four different time points representing four stages of disease development namely, before inoculation (0 days after inoculation, DAI), at the time of cortex infection (3 DAI), early onset (9 DAI), and later stage (20 DAI) of the disease.

### Microscopic Observation

For microscopic observation, the fresh tissue was fixed with fixed liquid for more than 36 h. The tissue was removed from the fixed liquid and trimmed with a scalpel in the ventilation cupboard; the trimmed tissue and the label were placed in the dehydration box; and the dehydration box was put into the dehydrator in order to dehydrate with gradient alcohol. The wax-soaked tissue was embedded in the embedding machine. Finally, placed the trimmed tissue wax block on the freezing table to cool at −20°C and sliced the modified tissue chip wax block on the paraffin slicer (with slice thickness: 4 μm). The tissue was flattened when the slice floated on the 40°C warm water of the spreading machine, and the tissue was picked up by the glass slides and baked in the oven at 60°C. After the water-baked dried wax was melted, it was taken out and stored at room temperature.

Toluidine blue staining was performed according to the following procedure: the slices were kept in xylene I for 20 min and in xylene II for 20 min. Then, they were kept in 100% ethanol I—100% ethanol II–75% ethanol, each step took around 5 min, and rinsed with tap water. Then the plant tissue slices were treated with toluidine blue for 2–5 min and rinsed with tap water. Then they were microscopically inspected, and according to the color of the tissues, checked for their differentiation. After tap water wash, they were dried in the oven. Finally, they were kept in xylene for 10 min, sealed with neutral gum, and followed microscope inspection, image acquisition, and analysis.

### RNA Extraction and Construction of cDNA Library

The RNA extraction and sequencing was performed by Beijing Biomarker Biotechnology Co., Ltd., Beijing, China. There, the RNA samples were assessed by 1% agarose gel electrophoresis for RNA degradation and impurities, Nanodrop 2000 for RNA purity and concentration, and Agilent 2100 RNA 6000 Nano Kit for RNA integrity (Agilent, Santa Clara, CA, United States). The Ribo-Zero rRNA Removal Kit (Epicentre, Madison, WI, United States) was used to remove rRNA. Sequencing libraries were generated using NEBNext Ultra^TM^ RNA Library Prep Kit for Illumina^®^ [New England Biolabs (NEB), Ipswich, MA, United States] following the recommendations of the manufacturer, and index codes were added to attribute sequences to each sample. mRNA was purified from total RNA using poly-T oligo-attached magnetic beads. Fragmentation was carried out using divalent cations under elevated temperature in NEBNext First-Strand Synthesis Reaction Buffer (5×. First-strand cDNA was synthesized using random hexamer primer and M-MuLV Reverse Transcriptase). Second-strand cDNA synthesis was subsequently performed using DNA Polymerase I and RNase H. Remaining overhangs were converted into blunt ends *via* exonuclease/polymerase activities. After adenylation of 3′ ends of DNA fragments, NEBNext Adaptor with hairpin loop structure was ligated to prepare for hybridization. In order to select cDNA fragments, preferentially of 240 bp in length, the library fragments were purified with AMPure XP system (Beckman Coulter, Beverly, MA, United States). About 3 μl USER Enzyme (NEB) was used with size-selected, adaptor-ligated cDNA at 37°C for 15 min followed by 5 min at 95°C before PCR. PCR was performed with Phusion High-Fidelity DNA polymerase, Universal PCR primers and Index (X) Primer. Finally, PCR products were purified (AMPure XP system) and library quality was assessed on the Agilent Bioanalyzer 2100 system.

### RNA Sequencing, Data Preprocessing, and Analysis

Sequencing was done using the Illumina HiSeq^TM^ 2500 platform for paired-end sequencing. The raw data were cleaned by removing the adapter sequence, N-sequence, and low-quality reads. Then, Q20, Q30, GC contents, and sequence repetition levels were calculated. The obtained clean data were then compared with the reference genome of Chinese cabbage (version 1.5).^1^ Gene expression levels were estimated by fragments per kilobase of transcript per million fragments mapped (FPKM) (Florea et al., 2013). Differential expression analysis of two samples was performed using the edgeR (Wang et al., 2009). The false discovery rate (FDR) of <0.01 and fold change of ≥2 was set as the threshold for determining significant differential expression. The raw reads were further processed with a bioinformatic pipeline tool, BMKCloud.^2^ Co-expression network analysis using WGCNA version 1.61 software package in R software (Langfelder and Horvath, 2008). The interaction network of hub-genes in module was visualized using Cytoscape 3.7.1.

### Quantification of Phytohormone

The samples were ground with liquid nitrogen. About 80 ± 5 mg of finely ground samples were then taken in a 2-ml centrifuge tube prior to adding 50 μl of internal standard solution and 1 ml of acetonitrile aqueous solution (1% FA). The mixtures were mixed well by shaking for 2 min and left for 12 h at 4°C in the dark for extraction. After that the samples were centrifuged at 14,000 × *g* for 10 min, and 800 μl of the supernatant were blow dried with nitrogen prior to reconstituting with 200 μl of acetonitrile water (1:1, v/v). This was further centrifuged at 14,000 × *g* for 10 min, and the supernatant was taken for analysis. The samples were separated by Agilent 1290 Infinity LC ultra-high-performance liquid chromatography system and analyzed by mass spectrometry. Selective reaction/multiple reaction monitoring (MRM) technology mode was used to detect the ion pair to be tested. Data processing and analysis were conducted using the Thermo Scientific Xcalibur 2.1 software package (Thermo Fisher Scientific, San Jose, CA, United States). The contents of plant hormones in the samples were estimated based on the standard curve.

## Results and Discussion

### Phenotypic and Cytological Observation of R- and S-Lines

**FIGURE 1:**
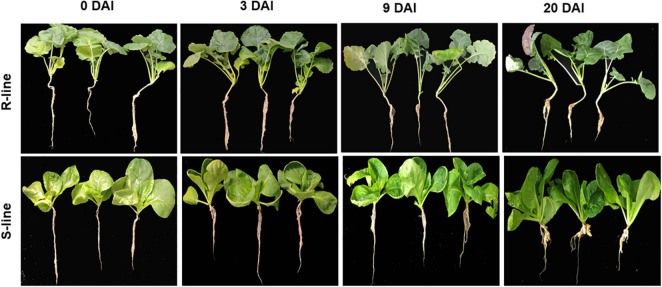
Clubroot symptoms in the roots of S- and R-lines at 0, 3, 9, and 20 days after inoculation (DAI) with race 4 strain of *Plasmodiophora brassicae*.

Upon inoculation, no obvious clubroot symptom was observed in both the R-line “BrT24” and S- line “Y510-9” till 9 DAI. At 20 DAI, conspicuous irregular swelling was observed in the roots of S-line, whereas no signs of swelling were found in the roots of R-line (Figure 1). These results indicate that clubroot develops more rapidly and severely on the susceptible genotype.

**FIGURE 2:**
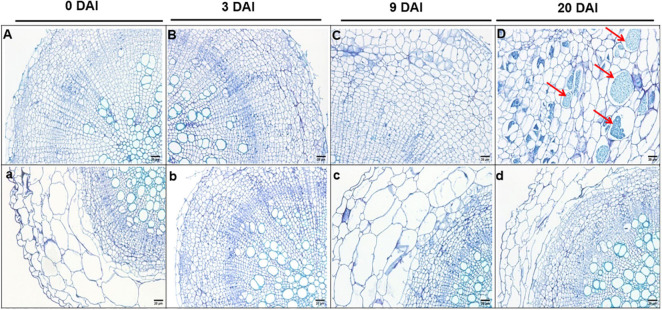
Cross sections (4 μm thick) of paraffin embedded roots of S-line **(A–D)** and R-line **(a–d)** at 0, 3, 9, and 20 DAI, respectively. Bar = 50 cm. DAI, days after inoculation.

Cross sections of paraffin-embedded roots indicate no obvious changes in the S- and R-lines at 3 DAI (Figures 2B,b) and there is no significant change compared with the uninoculated period (Figures 2A,a). At 9 DAI, irregularly enlarged and squeezed cells are observed in the roots of S-line (Figure 2C), whereas in the R-line, the root cells were not compressed or deformed (Figure 2C). At 20 DAI, S-line was severely infected by *P. brassicae*, R-line was slightly infested, and there is no obvious cell swelling and squeezing in R-line (Figure 2d). However, in S-line, the enlarged cells contain a large number of dormant spores, the root cortex cells are compressed by the enlarged cells, and the deformed cells are arranged disorderly (Figure 2D).

### Overview of the Transcriptome Profiles in R- and S-Lines at Different Time Points

**FIGURE 3:**
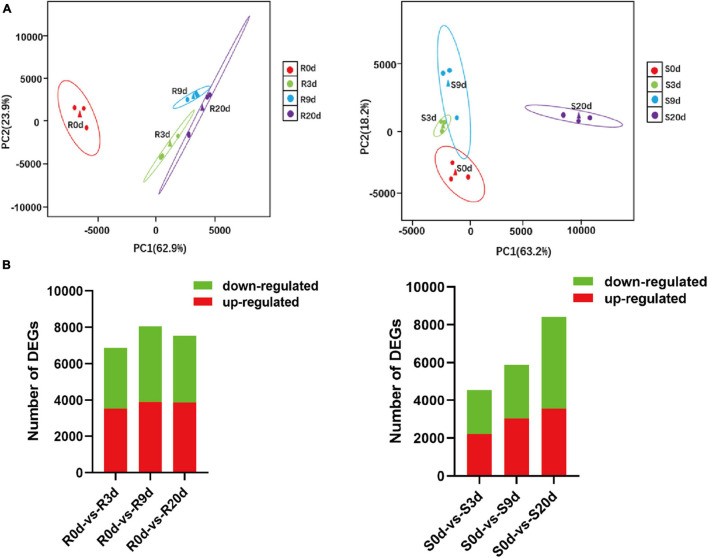
Overview of the transcriptomic responses in the roots of *Brassica rapa* upon inoculation with *P. brassicae*. PCA plots of the RNA-seq-derived transcriptomic responses **(A)** and numbers of significantly differentially expressed transcripts **(B)** in R-line and S-line at 0, 3, 9, and 20 DAI. DAI, days after inoculation; PCA, principal component analysis.

A total of 24 libraries from the three replicates of the root RNA samples of R- and S-line at 0, 3, 9, and 20 DAI were constructed and analyzed. A total of 291.23 Gb clean data (10.42 Gb per sample) was obtained. The Q30 values were all above 94.64%, indicating that the quality and accuracy of sequencing data were sufficient for subsequent analyses (Supplementary Table 1). Principal component analysis (PCA) plot of gene expression levels of the samples of R-line indicated that samples of 3, 9, and 20 DAI clustered far from the samples of 0 DAI (separated by PC1). In S-line, the samples of 0, 3, and 9 DAI were clustered far from the samples of 20 DAI (separated by PC1). This indicated that the S-line did not respond promptly to the root disease on the 3 and 9 DAI (Figure 3A).

A total of 41,087 transcripts were detected across all samples (Supplementary Table 2). Compared to 0 DAI, at 3 DAI, 3507, and 3,362 genes were upregulated and downregulated, respectively, in R-line, whereas 2,204 and 2,356 genes were upregulated and downregulated, respectively, in S-line (Figure 3B). At 9 DAI, 3,912 genes were upregulated, and 4,151 genes were downregulated in R-line, whereas only 3,045 genes were upregulated and 2,843 genes were downregulated in S-line. At 20 DAI, 3,880 and 3,660 genes were upregulated and downregulated in R-line, whereas 3,558 and 4,866 genes were upregulated and downregulated in S-line (Figure 3B). The FPKM values of the genes are shown in Supplementary Table 3.

Overall, the change of gene expression patterns of 12 selected differentially expressed genes (DEGs) (Supplementary Table 4) were examined by quantitative real-time PCR (qRT-PCR) to validate the RNA-seq results. The high correlation coefficients of log2 fold changes obtained from RNA-seq and qRT-PCR results suggested that the RNA-seq data in this study is reliable (Supplementary Figure 1).

### Contrasting Patterns of Gene Expression Between R- Versus S-Line at the Same Time Point

The genes that are contrastingly expressed in R- and S-lines may play key roles in plants responses toward *P. Brassicae* infection. Genes downregulated in R-line and upregulated in S-line could be susceptibility factors, whereas genes upregulated in R-line and downregulated in S-line could be involved in resistance. Such genes are good candidates for gene editing-based mutagenesis to either validate gene function or increase host resistance.

At 3 DAI, 29 genes were significantly downregulated in R-line but upregulated in S-line. On the contrary, 60 genes were upregulated in R-line and downregulated in S-line at 3 DAI (Supplementary Figure 2A). At 9 DAI, 30 genes were upregulated in the S-line and downregulated in R-line, 35 genes were upregulated in R-line and downregulated in S-line at 9 DAI (Supplementary Figure 2B). At 20 DAI, 209 genes were upregulated in S-line and downregulated in R-line, 868 genes were upregulated in R-line and downregulated in S-line at 20 DAI (Supplementary Figure 2C).

### Relative Differentially Expressed Genes Between R- and S-Lines

In order to exclude the background difference between resistant and susceptible materials, we analyzed the relative transcriptional changes over time. In R- or S-lines, DEGs at 3, 9, and 20 DAI were identified by comparing the expression levels at 3 DAI with those at 0 DAI, the level at 9 DAI with those at 3 DAI, and the level at 20 DAI with those at 9 DAI (Supplementary Figure 3A). In R-line, 3,507, 1,119, and 136 DEGs were upregulated, and 3,362, 1,391, and 67 DEG were downregulated at 3, 9, and 20 DAI, respectively. In S-line, 2,204, 1,285, and 2,648 DEGs were upregulated, and 2,356, 589, and 4,549 DEGs were downregulated at 3, 9, and 20 DAI, respectively (Supplementary Figure 3A). Compared with S-line, 2,475, 2,182, and 4,292 relative DEGs were identified in R-line at 3, 9, and 20 DAI, respectively (Supplementary Figure 3B). The above results indicate that the number of DEGs in 3 DAI of R-line is relatively large compared with other time points, indicating that 3 DAI is the key time point for R-line to respond to clubroot. It also indicates that the R-line activates the defensive response earlier compared to that of S-line.

### Weighted Gene Co-expression Network Analysis

**FIGURE 4:**
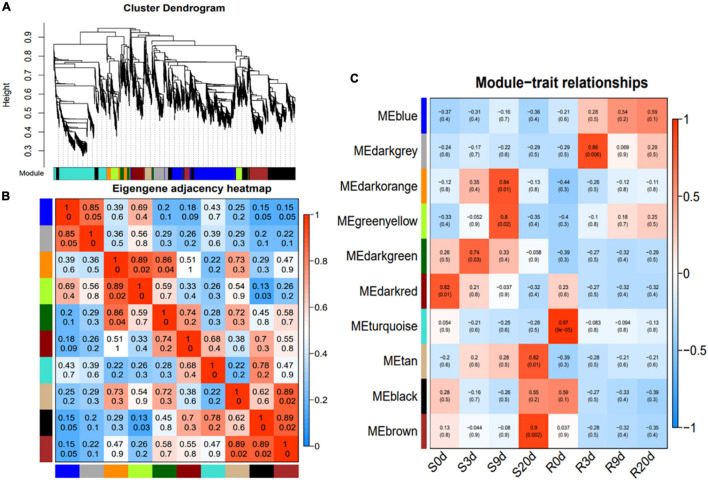
Weighted gene co-expression network analysis (WGCNA) of the relative DEGs in the R- and S-lines at 0, 3, 9, and 20 DAI. **(A)** Average linkage hierarchical clustering dendogram of the genes. Modules, designated by color code, are the branches of the clustering tree. **(B)** Eigengene adjacency heatmap of each module representing the correlation between the modules. **(C)** Correlation analysis between module and *P. brassicae* infected samples of R- and S-lines. The number on each cell is the correlation coefficient between each module gene and infected sample, and the number below is the corresponding *p*-value. DAI, days after inoculation; DEG, differentially expressed gene.

The weighted gene co-expression network analysis (WGCNA) of 6,092 valid genes (Supplementary Table 3) was carried out. The genes were assigned into 10 distinct modules (Figure 4A). A module is a cluster of highly interconnected genes with similar expression changes in a physiological process. The modules were then associated with each of the samples, which identified seven modules namely, blue, darkgray, darkorange, greenyellow, darkgreen, tan, and brown to be highly correlated with R- or S-line (Figures 4B,C). The Kyoto Encyclopedia of Genes and Genomes (KEGG) enrichment analysis of these seven modules revealed that the genes of three modules (blue, darkgray, and greenyellow) highly significantly enriched six pathways namely, Plant–pathogen interaction, Plant hormone signal transduction, Phenylalanine metabolism, Phenylpropanoid biosynthesis, Taurine and hypotaurine metabolism and Nitrogen metabolism (Table 1).

**TABLE 1:** Kyoto Encyclopedia of Genes and Genomes (KEGG) enrichment analysis of the genes of seven modules that were significantly associated with R- or S-line in weighted gene co-expression network analysis (WGCNA) revealed the significantly enriched pathways.

Module	ko_id	Description	*p*-Value	Associated sample
Blue	ko00360	Phenylalanine metabolism*	1.33E-06	R3d, R9d, and R20d
	ko00940	Phenylpropanoid biosynthesis*	5.31E-06	
	ko04626	Plant–pathogen interaction*	9.85E-06	
	ko00430	Taurine and hypotaurine metabolism*	9.99E-05	
Darkgray	ko04075	Plant hormone signal transduction*	0.000138234	R3d
	ko00410	beta-Alanine metabolism	0.000676243	
	ko00561	Glycerolipid metabolism	0.003039608	
Darkorange	ko00591	Linoleic acid metabolism	0.001143633	S9d
	ko00650	Butanoate metabolism	0.004553007	
	ko00250	Alanine, aspartate and glutamate metabolism	0.025391	
Greenyellow	ko00940	Phenylpropanoid biosynthesis*	2.82E-07	S9d
	ko00910	Nitrogen metabolism*	0.000527024	
	ko04626	Plant–pathogen interaction*	0.008774838	
	ko00960	Tropane, piperidine, and pyridine alkaloid biosynthesis	0.045914204	
	ko00220	Arginine biosynthesis	0.380775512	
Darkgreen	ko04144	Endocytosis	0.028533145	S3d
Tan	ko00480	Glutathione metabolism	1.34E-05	S20d
Brown	ko00910	Nitrogen metabolism	0.009827585	S20d
	ko00600	Sphingolipid metabolism	0.012087052	

The six highly significantly enriched pathways are indicated with an asterisk (*). KEGG, Kyoto Encyclopedia of Genes and Genomes.

**FIGURE 5:**
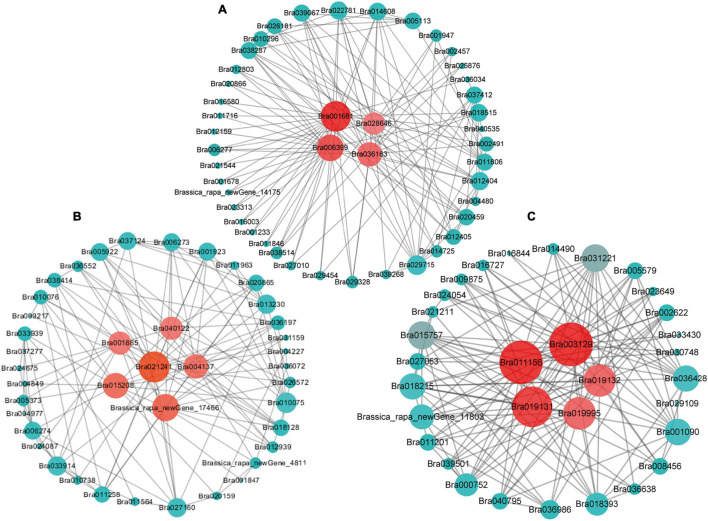
The hub genes identified by the gene co-expression network of “blue” **(A)** “darkgray” **(B)**, and “greenyellow” **(C)** modules. The hub genes are highlighted by red circles in the center.

Gene interaction network analysis of the genes of these three modules was done using Cytoscape 3.7.1, which revealed a total of 15 hub genes (Figures 5A–C and Table 2). Among these, the notable genes include cleavage site for pathogenic type III effector avirulence factor avr 4 (*RIN4*), auxin-responsive protein gene (*IAA16*) of plant hormone signal transduction pathway, universal stress protein family (*Bra019995*), peroxidase 44 (*PER44*) under phenylpropanoid biosynthesis pathway, protein detoxification 9 (*DTX9*) gene, HSF-type DNA-binding gene (*HSFA6b*), probable E3 ubiquitin-protein ligase (*ARI16*), b-cell receptor-associated 31-like protein (*Bra001681*), glutamate decarboxylase 1 (*GAD1*), glutamate decarboxylase 2 (*GAD2*), and s-adenosylmethionine synthetase (*SAMS3*) in cysteine and methionine metabolism, carbohydrate-binding protein of the ER (*SIRK*), and *Brassica_rapa_new Gene_17466*.

**TABLE 2:** Detailed information statistics of the identified hub genes.

Module	Gene	Annotation	KEGG pathway
Blue	Bra001681	B-cell receptor-associated 31-like protein	–
	Bra028646 (*ARI16*)	Probable E3 ubiquitin-protein ligase	–
	Bra006399 (*GAD1*)	Glutamate decarboxylase	Alanine, aspartate and glutamate metabolism (ko00250)
	Bra036163 (*RIN4*)	Cleavage site for pathogenic type III effector avirulence factor Avr	–
Darkgray	Bra021241 (*SAMS3*)	S-adenosylmethionine synthetase	Cysteine and methionine metabolism (ko00270)
	Bra040122 (*IAA16*)	Auxin-responsive protein	Plant hormone signal transduction (ko04075)
	Bra001885 (*HSFA6b*)	HSF-type DNA-binding	–
	Bra015208 (*DTX9*)	Protein DETOXIFICATION 9	–
	Bra004137 (*GAD2*)	Glutamate decarboxylase 2	Alanine, aspartate and glutamate metabolism (ko00250)
	*Brassica_rapa*_new Gene_17466	–	–
Greenyellow	Bra003129 (*CYP71B5*)	Cytochrome P450	Limonene and pinene degradation (ko00903)
	Bra011156 (*SIRK)*	Carbohydrate-binding protein of the ER	–
	Bra019131 (*PER44*)	Peroxidase	Phenylpropanoid biosynthesis (ko00940)
	Bra019132 (*PER44*)	Peroxidase	Phenylpropanoid biosynthesis (ko00940)
	Bra019995	Universal stress protein family	–

*RIN4* is a negative regulator of PTI, since it interacts with *Pseudomonas syringae* type III effector molecules, and its overexpression has been shown to increase PAMP-triggered cell wall thickening in *Arabidopsis* (Mackey et al., 2002; Hou et al., 2011). This *RIN4* may have a similar role in CR in *B. rapa*. *IAA16* promotes root responsiveness to ABA and is involved with growth of roots, cotyledons, and hypocotyls of *Arabidopsis* seedlings (Schmid et al., 2005; Rinaldi et al., 2012). Peroxidase-generated apoplastic reactive oxygen species (ROS) were found to compromise cuticle integrity and contribute to damage-associated molecular pattern (DAMP)-elicited defenses in *Arabidopsis*. It will be interesting to investigate in future if *PER44* and another detoxification-related gene, *DTX9*, identified as hub genes in our study, have any roles in resistance against CR (Mantas et al., 2016). Overexpression of glutamate decarboxylase conferred resistance to the northern root-knot nematode in tobacco (Mantas et al., 2016). The two hub glutamate decarboxylase genes, *GAD1* and *GAD2*, identified in our study, may have similar roles against clubroot. Overall, these hub genes may play pivotal roles in conferring resistance against clubroot disease in *B. rapa*.

### Functional Enrichment Analyses of Differentially Expressed Genes

**FIGURE 6:**
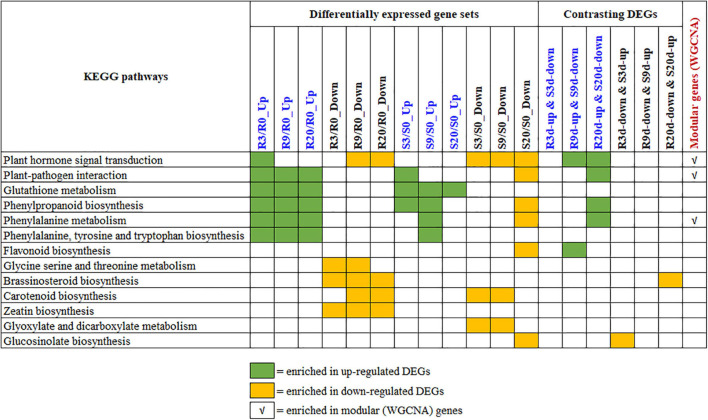
Summary of the key KEGG pathways that are significantly enriched by various differentially expressed gene (DEG) sets. The statistics of the pathway enrichment analyses for DEGs, contrasting DEGs, and modular genes are shown in Supplementary Figures 2, 4 and Table 1, respectively. The list of genes under each of these KEGG is shown in Supplementary Table 5. KEGG, Kyoto Encyclopedia of Genes and Genomes.

The KEGG enrichment analyses of the upregulated and downregulated DEGs in R- and S-lines (Supplementary Figure 4), contrasting DEGs between R- and S-lines (Supplementary Figure 2) and the seven modular genes (Table 1), were conducted to understand the biological mechanisms of CR. The key significantly enriched pathways of all these KEGG enrichment analyses are summarized in Figure 6 and the list of genes under each significant KEGG pathways are shown in Supplementary Table 5.

Overview of all the significantly enriched KEGG pathways revealed that the plant hormone signal transduction pathway is only enriched by the upregulated genes of R-line at 3 DAI (but not at 9 and 20 DAI). Contrastingly, this pathway was enriched by the downregulated genes of 9 and 20 DAI in R-line and all time points in S-line. This contrasting signal transduction in R- vs. S-lines clearly indicates that hormone signal transduction was activated and thereby, plants immune responses were initiated at an earlier stage of infection in R-line. Besides, enrichment of the pathway by upregulated DEGs of R-line and downregulated DEGs of S-line at 9 and 20 DAI indicate that hormone signaling based immune response is still in operation in R-line which is absent in S-line. This is also evident from the enrichment of the plant–pathogen interaction pathway by the upregulated DEGs at all time-points in R-line whereas only by the upregulated DEGs at 3 DAI in S-line. This indicates that plants interaction with the pathogen is occurring at all times in R-line whereas this immune response is inhibited at later time points in S-line. To exclude the background difference, we performed the KEGG enrichment analysis of the relatively DEGs. For relative DEGs, the genes involved in CR regulation are indeed enriched in plant–pathogen interaction, plant hormone signal transduction, and phenylpropanoid biosynthesis (data not shown).

Brassinosteroid biosynthesis and Zeatin biosynthesis were enriched by the downregulated DEGs of R-line at different time points. These pathways were not enriched by the up- or downregulated DEGs of S-line. Isoquinoline alkaloid biosynthesis is only significantly enriched in genes that are upregulated in R-line and downregulated in S-line by 3 DAI.

Contrastingly, glyoxylate and dicarboxylate metabolism pathway is enriched by the downregulated DEGs of S-line at initial stages of infection, and GSL biosynthesis is only enriched by the downregulated DEGs of S-line at a later stage of infection. Glyoxylate and dicarboxylate metabolism is known to play important functions in the detoxification during stress and contribute to redox balance in plants (Allan et al., 2009). GSLs, a group of sulfur-containing plant secondary metabolites found in the cruciferous family, play an important role in the resistance of *Brassica* root disease (Ludwig-Müller et al., 2009; Zhang et al., 2016; Li et al., 2019).

Upregulated DEGs (at all time points) of both R- and S-lines are enriched in glutathione metabolism pathway, whereas phenylpropanoid biosynthesis, phenylalanine metabolism, and phenylalanine, tyrosine, and tryptophan biosynthesis pathways were enriched by the upregulated DEGs of all time points in R-line and by the upregulated DEGs of initial time points and downregulated DEGs of later time points in S-line (Figure 6). This indicates that these pathways can increase the resistance during the secondary infection process of clubroot. A similar observation was also observed by Irani et al. (2019).

### Response of Phytohormone-Related Genes and Hormone Contents to *Plasmodiophora brassicae* in Different Periods

The pathway enrichment analysis of DEGs showed that “Plant hormone signal transduction,” “Alanine, aspartate and glutamate metabolism,” and “Phenylpropanoid biosynthesis” pathways are involved in the immune response to *P. brassicae*. Phytohormones play an important role in the different growth and development processes of plants and various biotic and abiotic stress responses. Early studies have shown that different pathogens infect plants can cause changes in the levels of different phytohormones (Adie et al., 2007; Robert-Seilaniantz et al., 2007). In order to further explore the role of plant hormones in defense against *P. brassicae*, we quantified the contents of phytohormone and examined the changes in expression of genes of related pathways in the contrastingly resistant lines at different time points after inoculation, which is discussed thoroughly in the following section.

#### Auxin Responsive Protein Gene and Cytokinin Play Key Roles in the Formation of Clubroot in S-Line and Are Inhibited in Mid- and Later Stages of Infection in R-Line

**FIGURE 7:**
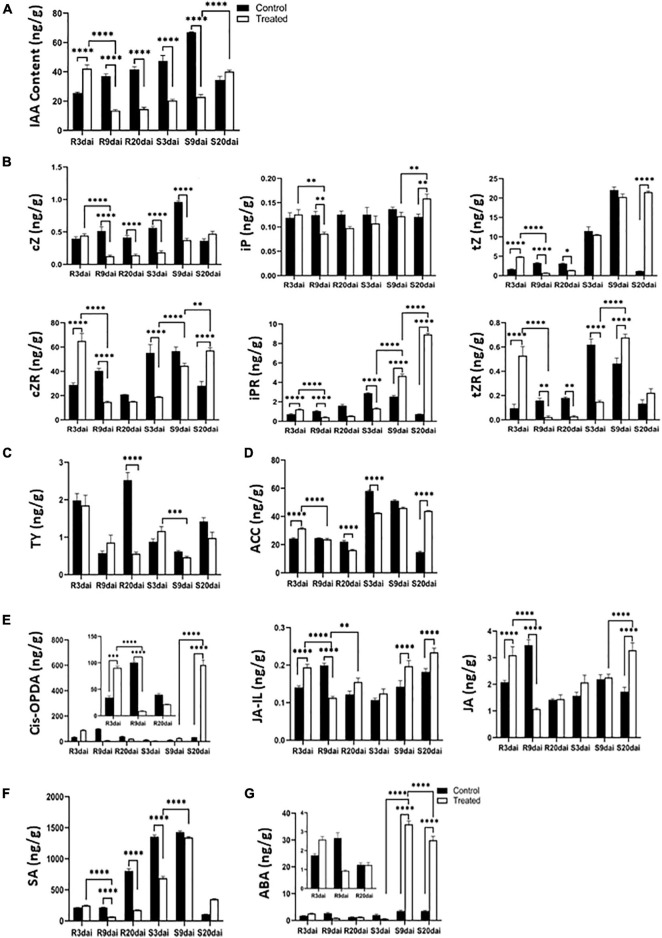
The contents of different phytohormones in the roots of R- and S-lines at 0, 3, 9, and 20 days after inoculation with *Plasmodiophora brassicae*. **(A)** IAA; **(B)** CTK; **(C)** BR; **(D)** ET; **(E)** JA; **(F)** SA; **(G)** ABA. ^****^, ^∗∗∗^, ^∗∗^, and ^∗^ indicate *p* < 0.0001, *p* < 0.001, *p* < 0.01, and *p* < 0.05, respectively.

In the later stages of clubroot infection, the S-line increases the rate of cell division in roots leading to hypertrophy of the infected roots. Subsequently, *P. brassicae* grows, and the host cell enlarges accordingly. Cell division is known to be influenced by auxin and CTK (Siemens et al., 2006). Cell enlargement, on the other hand, is only related to higher level of auxin (Grsic-Rausch et al., 2000; Devos et al., 2005; Päsold et al., 2010). Several studies have shown that auxin increases promote plant disease symptoms, and blocking the auxin response has been shown to increase plant resistance (Yamada, 1993; Chen et al., 2007; Dong et al., 2007; Navarro et al., 2016). In our study, the Indoleacetic acid (IAA) content increased significantly in R-line at 3 DAI but decreased significantly in S-line compared to respective uninoculated plants (Figure 7A). At 9 and 20 DAI, the IAA content decreased significantly in R-line and decreased in S-line at 9 DAI (Figure 7A), but increased at 20 DAI, indicating that IAA played an important role in the formation of plant root galls in S-line. On the other hand, the decrease of IAA content with the increase of infection period may have an inhibitory effect on the disease in R-line.

**FIGURE 8:**
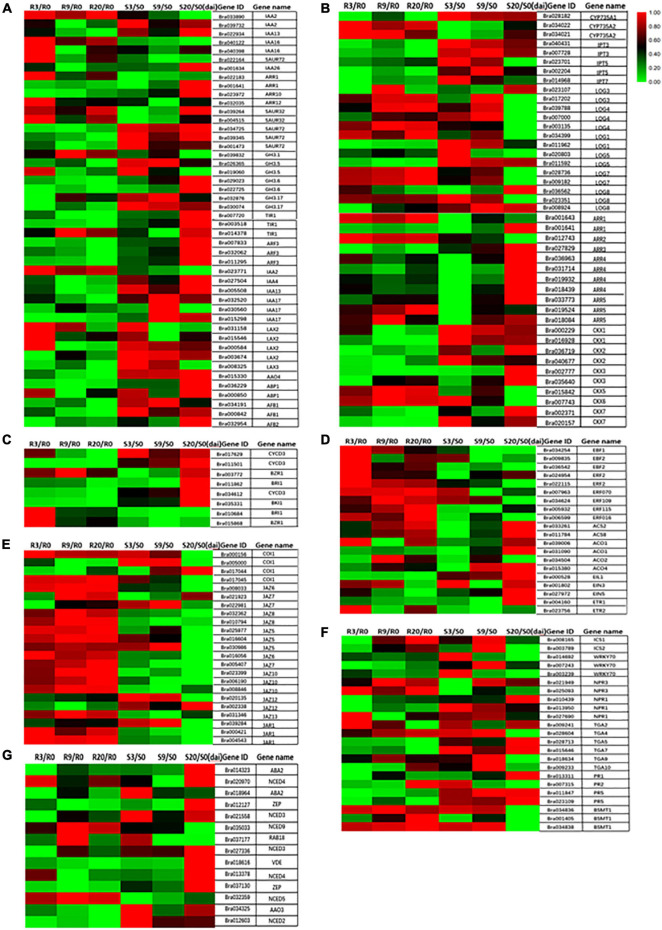
Heat maps showing differential expression of different hormone-related genes at 0, 3, 9, and 20 days after inoculation. **(A)** IAA; **(B)** CTK; **(C)** BR; **(D)** ET; **(E)** JA; **(F)** SA; **(G)** ABA.

The transcriptome sequence data clearly show that most of IAA pathway-related genes were upregulated in R-line at 3 DAI (e.g., *IAA16*, *SAUR32*, *ARR12*, and *LAX2*) and were downregulated at 9 and 20 DAI (Figure 8A). Previous studies have shown that the auxin conjugate synthetases (*GH3*) gene is upregulated when the auxin level is higher (Jahn et al., 2013). This gene can regulate IAA homeostasis, thereby contributing to plant disease symptoms to a certain extent (Ludwig-Muller, 2014). In addition, the auxin binding protein 1 *(ABP1*) reduces the symptoms of *P. brassicae* through the activation of potassium channels (Jahn et al., 2013). In our study, most of the genes of the *GH3* family were downregulated during clubroot infection in the R-line and upregulated in the S-line (Figure 8A), but most of them are upregulated in R-line compared to the S-line (Unpublished data). Two different classes of auxin receptors, the *TIR* family and *ABP1* in *B. rapa* except for one copy of *TIR1* are upregulated in R-line, most of them are downregulated in R-line (Figure 8A).

The quantitative measurements of CTKs indicated that isopentenyladenine (iP) were the major active CTKs found in both uninfected and infected tissues. The content of iP did not show much variation in the control and infected tissues of R- and S-lines (Figure 7B). The contents of *trans*-zeatin (tZ), *cis* zeatin (cZ), *trans*-zeatin riboside (tZR), N6-isopentenyladenosine (iPR), and *cis*-zeatin riboside (cZR) significantly increased at 3 DAI, which then significantly decreased at 9 and 20 DAI compared to respective control samples in R-line (Figure 7B). The overall pattern was very clearly repressive as the majority of CTK metabolites showed significant reductions in expression in infected tissue. Bishopp et al. (2011) found that the concentration of active CTKs, CTK ribosides, and N-glucosides was significantly reduced at both 16 and 26 DPI, and the reduction in tZ was much greater than iP at 16 DPI. This is consistent with acropetal tZ transport *via* the xylem and basipetal iP transport *via* the phloem. The impact on tZ is likely to have been greater as xylogenesis is strongly repressed in the root during gall formation. Devos et al. (2005) also found increased zeatin content in the early stage of infection, which decreased in the later stage. However, they did not distinguish between *cis* and *trans*. Devos et al. (2006) also measured the CTK content of infected *Arabidopsis* tissues at 4 DAI and found that zeatin and iP increased slightly at this time, which may indicate that CTK played a role in the early stages of *P. brassicae.* It is also possible that the early vascular bundles have not been formed, and the content of CTK is relatively high. As the later vascular bundles begin to form, the CTK content decreases, which is beneficial to the development of vascular bundle tissues. These are consistent with our research results.

The expressions of most of the genes associated with CTK biosynthesis were repressed in R-line with the increase in infection period (Figure 8B). The expressions of the adenine isopentenyl transferase genes *IPT3* and *IPT5* were greatly reduced. Reduced expression was also observed for *CYP735A1* and *CYP735A2* genes, which convert iP nucleotides to the corresponding tZ molecules and *LOG* genes associated with the production of active CTK species from CTK ribosides. Most downstream response regulators (ARRs) and CTK oxidases/dehydrogenases were strongly inhibited during infection. Malinowski et al. (2016) also observed downregulation of two of seven known putative CTK biosynthesis genes and CTK oxidases/dehydrogenases 1 and 6 (*AtCKX1* and *AtCKX6*). A reduction of the degradation capacity of CTKs by inhibition of specific *CKX* genes could in turn result in increased content of CTKs, especially at the sites where plasmodia produce CTKs. In *B. rapa*, an induction of isopentenyl transferase genes involved in the *de novo* biosynthesis of CTKs in clubroots has been described (Ando et al., 2005). Gall formation is reduced in plants constitutively expressing CTK oxidase as in these plants the reduction in the CTK content will slow the formation of the vascular cambium (VC) and the formation of gall (Malinowski et al., 2016). This indicated that the active CTK signaling is necessary for gall development. Our results are in broad agreement with previous studies in *Arabidopsis* and other *Brassicas* where CTK metabolism has been reported to be repressed at the onset of gall formation (Ando et al., 2005; Siemens et al., 2006; Agarwal et al., 2011).

#### Brassinosteroid and Ethylene Play an Important Role in R-Line Resistance

The content of typhasterol 8 (TY) in R-line decreased with the increase of inoculation time (Figure 7C). BR-related genes are also mostly repressed in R-line and induced in S-line by *P. brassicae* (Figure 8C). Contrastingly, the TY content in the S-line was not significantly different from the respective control group (Figure 7C). This indicates that R-line may mediate resistance to *P. brassicae* through the inhibition of BR expression. Schuller et al. (2014) also found upregulation of BR signaling pathways in hosts containing large and small plasmodium, and they were treated with BR synthesis inhibitors and BR receptor mutants. It shows the synergistic effect of BR in the pathogenesis of clubroot disease, which is consistent with our research results.

Ethylene is a gaseous plant hormone with many functions including activation of defense genes. At present, it is generally accepted that ET works with JA in the activation of defenses against necrotrophic pathogens (Glazebrook, 2005). In our quantitative detection of plant hormones, 1-aminocylopropane-1-carboxylic acid (ACC) content showed a significant decrease at 3 and 9 DAI, and at 20 DAI, it showed a significant decrease compared to control in R-line. Compared to the control, in S-line, the ACC content was significantly decreased at 3 DAI, and significantly increased at 20 DAI (Figure 7D). Research shows that growth and seed production of ET response 1 (*etr1*), ET insensitive2 (*ein2*), and ET insensitive 3 (*ein3*/*eil1*) mutants were inhibited rather than promoted by *Piriformospora indica*, a growth-promoting fungus, indicating that the ET-signaling components might be required for a balance between the host and endophyte (Camehl et al., 2010). Among the ET receptor mutants, ET insensitive 4 (*ein4*) could target different signaling pathways, and thus *ein4* is slightly more resistant to clubroot disease than other receptor mutants (Hall and Bleecker, 2003).

In our study, *ETR1* was downregulated at 3, 9, and 20 DAI, *EIN3* was also downregulated at 20 DAI, and *EIN4* and *EIN5* were all downregulated at 9 DAI and 20 DAI in R-line (Figure 8D). In addition, three IEN3-binding F-box protein (EBF) genes, six ET response factor (*ERF*) genes, two 1-aminocyclopropane-1-carboxylate synthase (*ACS*) genes, and four 1-aminocyclopropane-1-carboxylate oxidase (*ACO*) genes are all upregulated at 3, 9, and 20 DAI relative to control in R-line (Figure 8D). Upregulation of the *ACO* transcript in infected roots compared to controls was confirmed in R-line (Figure 8D). This is in accordance with a decrease of ACC content in infected roots. Our research results prove that ET signaling is needed to restrict gall growth. However, the resistance mechanism of ET against *P. brassicae* needs further study.

#### Jasmonate Acid Content Is Repressed in R-Line, but Strongly Induced in S-Line

Based on the types of pathogen, hormonal pathways differentially regulates plant’s responses toward different pathogens (Thomma et al., 1998; Oliver and Ipcho, 2004; Glazebrook, 2005). SA-mediated defense responses are generally directed against biotrophs/hemibiotrophs such as *powdery*- and *downy-mildew*, whereas JA/ET-dependent defense responses generally inhibit necrotrophs including *Alternaria*, *Pythium*, and *Botrytis* (Thomma et al., 1998; Glazebrook, 2005). In our study, the content of JA precursor (*cis*-OPDA), JA-bound (jasmonoyl-L-isoleucine, JA-Ile), and free JA content in the R-line showed a significant increase in the 3 DAI compared to the control, which then sharply decreased at 9 DAI. It returned to the same level as the control at 20 DAI (Figure 7E). Contrastingly, in S-line, compared to the control, *cis*-OPDA and JA extremely increased at 20 DAI, and JA-Ile showed a significant increase in both 9 and 20 DAI (Figure 7E). Previous studies have found that in the susceptible species of *Arabidopsis*, the infection of *P. brassicae* can cause the accumulation of JA, and then mediate the content of aliphatic GSLs, which can promote the development of clubroot disease. Exogenous application of JA can also achieve the same effect. It is believed that JA can promote the development of *P. brassicae* in susceptible varieties, leading to host susceptibility (Xu et al., 2016; Li et al., 2018). JA-zim (JAZ domain) protein is a repressor of JA signal transduction, and it has been proved (*JAZ1* and *JAZ3*) to interact with *JIN1*/*MYC2* and inhibit the expression of JA response genes (Bari and Jones, 2009). JAZ protein acts as a JA co-receptor and transcriptional repressor of JA signaling in *Arabidopsis* (Kazan and Manners, 2012), and degradation of the JAZ protein allows transcription factors (such as *MYC2*) to activate the expression of JA response genes (Chini et al., 2007).

About 13 genes of 16 JAZ genes were upregulated in R-line at 3, 9, and 20 DAI (Figure 8E). Studies have found that the jar1 mutant, impaired in JA-Ile accumulation, exhibited heightened susceptibility to clubroot (Agarwal et al., 2011; Antoine et al., 2012). In our study, three copies of jasmonate resistant 1 (*JAR1*) genes were all upregulated compared to the control, suggesting that the JA response participated in basal defense, which is consistent with our observations (Figure 8E). Our research confirms the previous view that in the prevention of *P. brassicae*, the inhibition of JA helps to resist *P. brassicae*, and the external application of JA can help increase the susceptibility of plants (Lemarié et al., 2015).

#### Salicylic Acid Is Suppressed in R-Line

After infection with *P. brassicae*, the accumulation of SA in R-line was lower than that in S-line. In R-line, the SA content was significantly reduced compared to control at 9 and 20 DAI. In S-line, the SA content was significantly reduced compared to control at 3 DAI, and there was no significant change in 9 and 20 DAI (Figure 7F). The non-expressor of PR (*NPR*) genes are involved in regulating SA levels. *NPR1* is the main transducer of SA signals, interacting as co-factor of transcription factors to alter defensive response (Cao et al., 1997; Dong, 2004). *PR1* interacts specifically with TGA factors (TGACG sequence-specific binding proteins), binds to the promoter of an important defense marker *PR1* (Chen et al., 2016), and regulates its activation (Weigel et al., 2005). Two *NPR1* homologs, identified as key regulators of SA-mediated resistance in *Arabidopsis thaliana*, were downregulated at 3, 9, and 20 DAI compared to the control (Figure 8F). Isochorismate synthase 1 (*ICS1*) and *ICS2* are two genes redundantly involved in SA synthesis (Garcion et al., 2008). In our study, compared to control, *ICS1* and *ICS2* were upregulated in R-line at 9 and 20 DAI and were significantly upregulated in S-line compared to control at 9 DAI (Figure 8F). Plant hormones can inactivate SA by converting it into methyl salicylate. Benzoic acid carboxyl methyltransferase (*BSMT1*) is a member of the SABATH methyltransferase gene family, and two of its three copies are significantly upregulated at all stages in R-line and were significantly upregulated in S-line at 3 and 9 DAI, and significantly downregulated at 20 DAI (Figure 8F). Studies have found that overexpression of *BSMT1* in *Arabidopsis* reduces SA levels by half, although this manipulation alone did not alter susceptibility to *P. brassicae* (Mohammad et al., 2018). This corresponds to our measured SA accumulation data.

The transcription factor *WRKY70* has also been shown to be a key regulator of the antagonistic response of SA and JA. It is regulated by *NPR1* and may bind to the promoter of the negative regulator of JA signal or induce a factor that blocks the positive regulator (Li et al., 2006). In our study, *WRKY70* was downregulated at all time points in the R-line compared to the control, which is consistent with a significant decrease in the SA content we measured (Figure 8F). However, it was inconsistent with previous findings that the SA-mediated defense response was strongly activated, whereas the JA pathway was inhibited, on the contrary (Gonzalez et al., 2020). *PR1*, *PR2*, and *PR5* are known to be SA responsive, and *PR2* and *PR5* have been shown to be induced during secondary infection by *P. brassicae* of partially resistant *A. thaliana* (Lemarié et al., 2015). In our study, two copies of the *PR5* gene were significantly upregulated at all time points in R-line and downregulated at all time points in S-line. *PR1* was also downregulated in R-line at all time points, upregulated at 9 and 20 DAI in S-line. *PR2* is significantly upregulated in R-line at 20 DAI and in S-line at 3 DAI and was downregulated at all other time points (Figure 8F). These data suggested a paradoxical situation where infection by the same single isolate would induce two different hormone responses depending on the plant genotype. Further research will be needed to confirm the role of SA in different plant genotypes.

#### ABA Plays an Important Role in Response to Abiotic Stress of S-Line in the Later Stages of Infection

The inhibition of water absorption and transportation due to the destruction of roots and vascular system during the secondary infection of *P. brassicae* triggers the drought stress response of plants (Ludwig-Müller et al., 2009). Under osmotic conditions such as high salinity and drought, ABA is known to stimulate short-term responses like closure of stomata, resulting in maintenance of water balance (Zhang et al., 1987) and longer-term growth responses through regulation of stress-responsive genes. The role of ABA in plant defense is very complex and differs in different types of plant–pathogen interactions.

We found that compared to the control, the ABA content of the inoculated *P. brassicae* in the R-line at 3 DAI was significantly increased, and there was no significant change at 9 and 20 DAI (Figure 7G). In S-line, the ABA content increased significantly compared to the control at 9 and 20 DAI. This is consistent with the results of previous studies that ABA has been shown to participate in the negative regulation of plant defense against various biological nutrients and necrotrophic pathogens (Audenaert et al., 2002; Adie et al., 2007; Asselbergh et al., 2008), and exogenous application of ABA enhanced the sensitivity of *Arabidopsis* plants to *Pst* (de Torres-Zabala et al., 2007). Probably that is why the ABA content has not changed significantly in R-line and in S-line, it has significantly increased. On the other hand, in S-line, as the infection time increases, the clubroot disease becomes severe, and ABA response to dehydration increases. Transcriptome data indicated that most ABA synthesis genes such as zeaxanthin epoxidase gene (*ZEP*), also known as low expression of osmotic stress-responsive gene 6 (*LOS6*)/*ABA1*, the aldehyde oxidase gene (*AAO3*), 9-*cis*-epoxycarotenoid dioxygenase gene (*NCED4 and NCED5*), and violaxanthin de-epoxidaseare (*VDE*) were upregulated by drought and salt stresses at 20 DAI in S-line, and only *NCED4*, *NCED5*, and *NCED9* responded in R-line. Our transcriptome data (Figure 8G) is consistent with the measured ABA content. Since these reactions only occur in later stages, these may be the result of dehydration rather than a signal of gall development.

**FIGURE 9:**
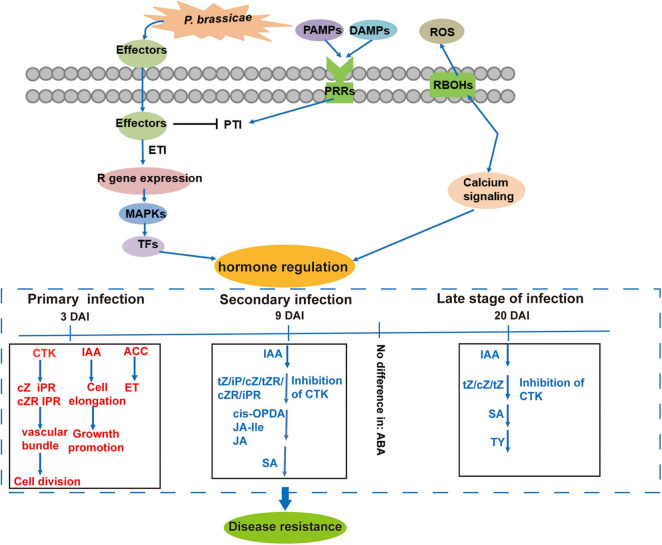
Proposed model of resistance response to *P. brassicae* in *B. rapa* of R-line based on hormonal regulation at different time points after pathogen inoculation in contrastingly resistant genotypes. Red and blue indicate up- and downregulated phytohormones, respectively.

Based on the summary of the changes in phytohormone signals and expressions of related genes, we propose a model of phytohormone-mediated defense mechanism induced by *P. Brassicae* (Figure 9). The inhibition of IAA, CTK, JA, and SA, and induction of BR and ET in the R-line and corresponding transcriptional changes in the related genes play important roles in the immune response of plants against clubroot.

## Conclusion

This study describes metabolome profiling and hormone-related transcriptomic responses of CR in *B. rapa*. The primary response of *P. brassicae* infection involves several pathways including plant hormone signal transduction and plant–pathogen interactions. Our study shows the importance of phytohormone in triggering immune responses and proposes a model of plants’ immune response against clubroot based on the observed changes in hormonal contents and expressions of related genes in *B. rapa*. Our findings will be helpful in enhancing our understanding of the mechanism of CR and will open up new research avenues for improving resistance of clubroot in future.

## Data Availability Statement

The original contributions presented in the study are publicly available. This data can be found here: National Center for Biotechnology Information (NCBI) BioProject database under accession number PRJNA743585.

## Author Contributions

XW and FW conceived and designed the experiments. YL, SY, and ZW performed the experiments. GS, HS, and ZX analyzed the data. YaZ, YY, and BT prepared the figures and tables. XW drafted the manuscript. YiZ, XZ, and MH critically revised the manuscript. All authors contributed to the article and approved the submitted version.

## Conflict of Interest

The authors declare that the research was conducted in the absence of any commercial or financial relationships that could be construed as a potential conflict of interest.

## Publisher’s Note

All claims expressed in this article are solely those of the authors and do not necessarily represent those of their affiliated organizations, or those of the publisher, the editors and the reviewers. Any product that may be evaluated in this article, or claim that may be made by its manufacturer, is not guaranteed or endorsed by the publisher.

## Footnotes

1
http://brassicadb.cn/#/Download/

2
http://www.biocloud.net
