# Supplementary material for: Root Transcriptome and Metabolome Profiling Reveal Key Phytohormone-Related Genes and Pathways Involved Clubroot Resistance in Brassica rapa L
Source: Front Plant Sci. 2021 Dec 15;12:759623. doi: 10.3389/fpls.2021.759623 (PMC8715091; doi:10.3389/fpls.2021.759623)
Supplement: Supplementary file 5 [file Data_Sheet_1.docx]

**Supplementary Information**

**Root Transcriptome and Metabolome Profiling Reveal Key** **Phytohormone-related Genes and Pathways involved Clubroot Resistance in *Brassica rapa* L.**

Xiaochun Wei^1,2†^, Yingying Zhang^1,2†^, Yanyan Zhao^1^, Zhengqing Xie^2^, Shuangjuan Yang^1^, Gongyao Shi^2^, Mohammad Rashed Hossain^3^, Yanyan Lv^1,2^, Zhiyong Wang^1^, Baoming Tian^2^, Henan Su^1^, Fang Wei^1,2*^, Xiaowei Zhang^1*^and Yuxiang Yuan^1*^

^1^Institute of Horticulture, Henan Academy of Agricultural Sciences, Graduate T &R Base of Zhengzhou University, Zhengzhou, Henan 450002, China.

^2^Henan International Joint Laboratory of Crop Gene Resources and Improvement, School of Agricultural Sciences, Zhengzhou University, Zhengzhou, Henan 450001, China.

^3^Department of Genetics and Plant Breeding, Bangladesh Agricultural University, Mymensingh 2202, Bangladesh.

†Equal contribution

*Correspondence: Fang Wei, Email: fangwei@zzu.edu.cn; Tel: (+86)371-67785055; Xiaowei Zhang, Email: xiaowei5737@163.com; Tel: (+86)371-65719797; and Yuxiang Yuan, Email: yuxiangyuan126@126.com

**Supplementary Tables:**

**Table S1.** Output of transcriptome sequencing data of each sample in susceptible and resistant materials

| Samples | ReadSum | BaseSum | GC(%) | N(%) | Q20(%) | Q30(%) |
| --- | --- | --- | --- | --- | --- | --- |
| S0 d-1 | 43687683 | 12992589800 | 44.47 | 0 | 98.81 | 95.91 |
| S0 d-2 | 36222954 | 10791042202 | 44.41 | 0 | 98.63 | 95.52 |
| S0 d-3 | 38153619 | 11393306018 | 45.04 | 0 | 98.75 | 95.77 |
| S3 d-1 | 37073547 | 11046164826 | 45.61 | 0 | 98.83 | 95.88 |
| S3 d-2 | 43502372 | 12997678666 | 45.12 | 0 | 98.76 | 95.77 |
| S3 d-3 | 41694521 | 12463981512 | 45.11 | 0 | 98.8 | 95.82 |
| S9 d-1 | 41292380 | 12335474038 | 45.36 | 0 | 98.83 | 95.87 |
| S9 d-2 | 43556647 | 13013668420 | 45.29 | 0 | 98.8 | 95.78 |
| S9 d-3 | 40088870 | 11960381324 | 44.57 | 0 | 98.83 | 95.95 |
| S20 d-1 | 38775109 | 11549910124 | 46.16 | 0 | 98.84 | 96.1 |
| S20 d-2 | 40706182 | 12152055466 | 46.88 | 0 | 98.75 | 95.88 |
| S20 d-3 | 40763538 | 12127826642 | 46.07 | 0 | 98.81 | 95.92 |
| R0 d-1 | 37027373 | 11020877038 | 45 | 0 | 98.78 | 95.83 |
| R0 d-2 | 39715704 | 11871769898 | 44.92 | 0 | 98.84 | 96.01 |
| R0 d-3 | 34830075 | 10422869144 | 44.47 | 0 | 98.45 | 95.14 |
| R3 d-1 | 36222729 | 10829322710 | 45.38 | 0 | 98.79 | 95.87 |
| R3 d-2 | 41440993 | 12367254910 | 45.29 | 0 | 98.77 | 95.8 |
| R3 d-3 | 46117129 | 13792068896 | 44.8 | 0 | 98.71 | 95.68 |
| R9 d-1 | 38828652 | 11607613408 | 45.34 | 0 | 98.87 | 96.02 |
| R9 d-2 | 39172056 | 11712615618 | 45.02 | 0 | 98.66 | 95.51 |
| R9 d-3 | 40064753 | 11947326948 | 45.54 | 0 | 98.83 | 95.96 |
| R20 d-1 | 44270517 | 13214582210 | 45.41 | 0 | 98.24 | 94.78 |
| R20 d-2 | 48085760 | 14365721726 | 45.07 | 0 | 98.33 | 94.97 |

**Table S2.** List of total 41088 transcripts detected across all samples.

[Supplied as separate excel sheet]

**Table S3.** List of differentially expressed genes along with their FPKM values upon infection with *P. brassiceae* in the R- and S-lines at 0, 3, 9 and 20 DAI.

[Supplied as separate excel sheet]

**Table S4.** Gene-speciﬁc primers sequences of qRT-PCR

| Gene | Forward primer（5' - 3'） | Reverse primer（5' - 3'） |
| --- | --- | --- |
| *Bra000375* | CGAGCTCAGATTCACCGAGG | GATGAACGGAGAGAGAGCGG |
| *Bra002153* | CGACCCAGAACCACTCTCAG | CGTGGTTGGACTATTGCGTC |
| *Bra003311* | GATTCGGATCCGGGTCTGTC | GTTGACGGCGAACGAGAAAC |
| *Bra003665* | GAGGGCTGAGGATGTTGAGG | GGCAAAACAATGTATACCTGAAGC |
| *Bra004125* | GGGTGAAATTGATGCAGGTCA | GGTGAGGTTCTGTGGGAAGG |
| *Bra006556* | GCAAGCTTGAGAAGTTGTGGG | AAACAGGAGTGACAATATCAGTTTT |
| *Bra007123* | AGGAGTCATGAAGTCTGGTGC | AGGTTAGCTGCTAGGGTTTGC |
| *Bra011955* | GAGCAACGAAGCGACTAACG | TCCATGTCCTGCACATAACCG |
| *Bra019409* | AGAAGCTGACCGGAGACTTTG | AGCATGAAGACAAAACCAGTTGC |
| *Bra021926* | GCAACAGGTTTGTTTCCTTGTC | ACACTGTTGCCTGCTCGTAA |
| *Bra023671* | ATTGGGATTGTGGGTCCTGC | TGCAAACGCAACTTCAACGA |
| *Bra025251* | TCACGCCGAGGATCTTGTTC | TTGCCACGCAAGAAACATCC |
| *Bra028685* | CGCCAAGGTAAACCTAAACTCC | TGGTAAACCGGAACCTCCAT |
| *Bra029142* | AAGGAGGGAAAGTTGCGGAG | CTAGAACCGGCCCTGCTAAC |
| *Bra037319* | ACAACGACCAGAGCATCCTG | AACAGAGCTGGAACCGAGTG |
| *Brassica_rapa_*  *newGene_22806* | AGTGGCCCACATGGAAAGTG | GCCTACGAGTACGAACTAGGG |
| *GAPDH-Putongqian* | GACTGGAGAGGTGGAAGAGCC | ACTGAAACATCAACGGTGGGA |

**Table S5.** List of genes enriched in the key significant KEEG pathways. The KEGG enrichment analysis are summarized in Figure 6.

[Supplied as separate excel sheet]

**Supplementary Figures:**


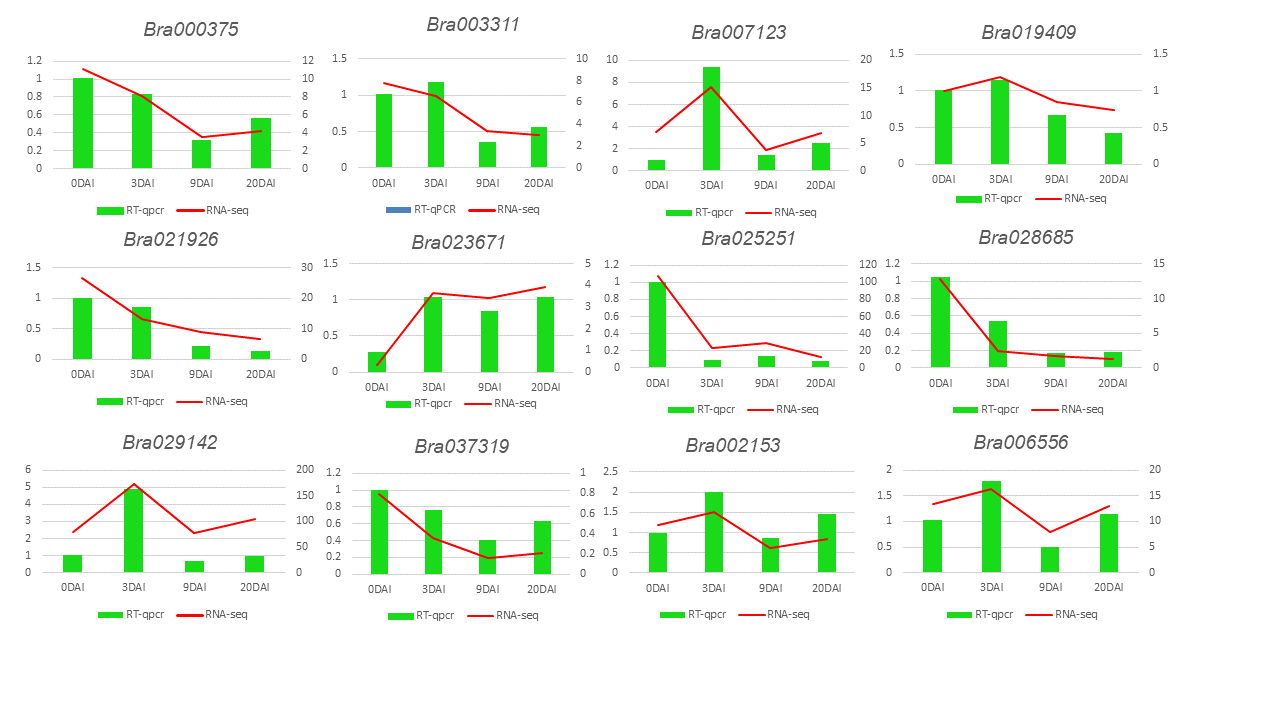


**Figure S1.** qRT-PCR verification of gene expressions of selected genes in susceptible and resistant lines.


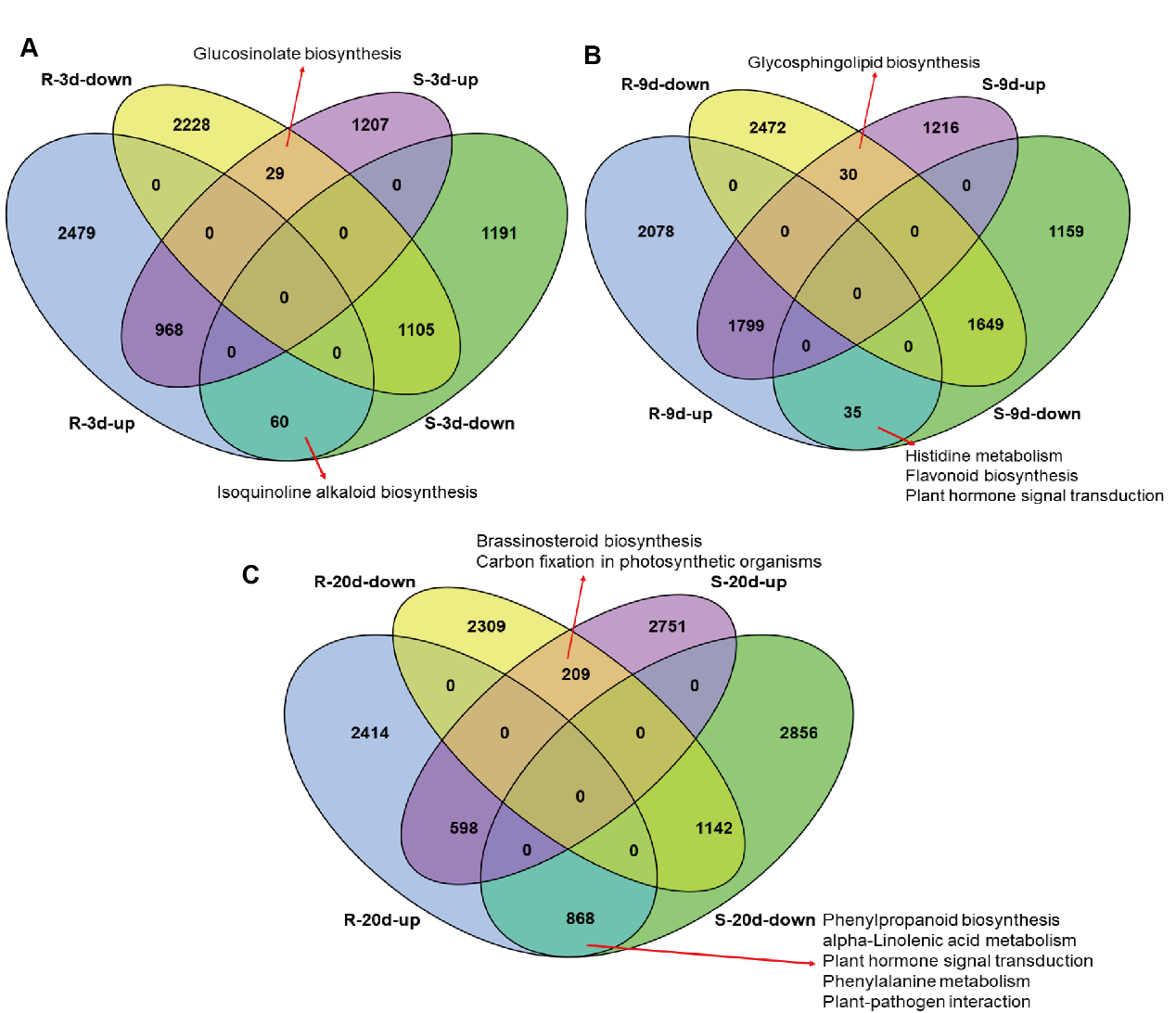


**Figure S2.**Venn diagram of DEGs 3, 9 DAI and 20 DAI. The diagram depicts the number of genes with common, opposite, and distinct expression patterns in‘R-line' and ‘S-line' in response to *P. brassicae*. Significantly enriched KEGG pathways of contrastingly regulated genes between R- and S-lines are shown with red arrows.


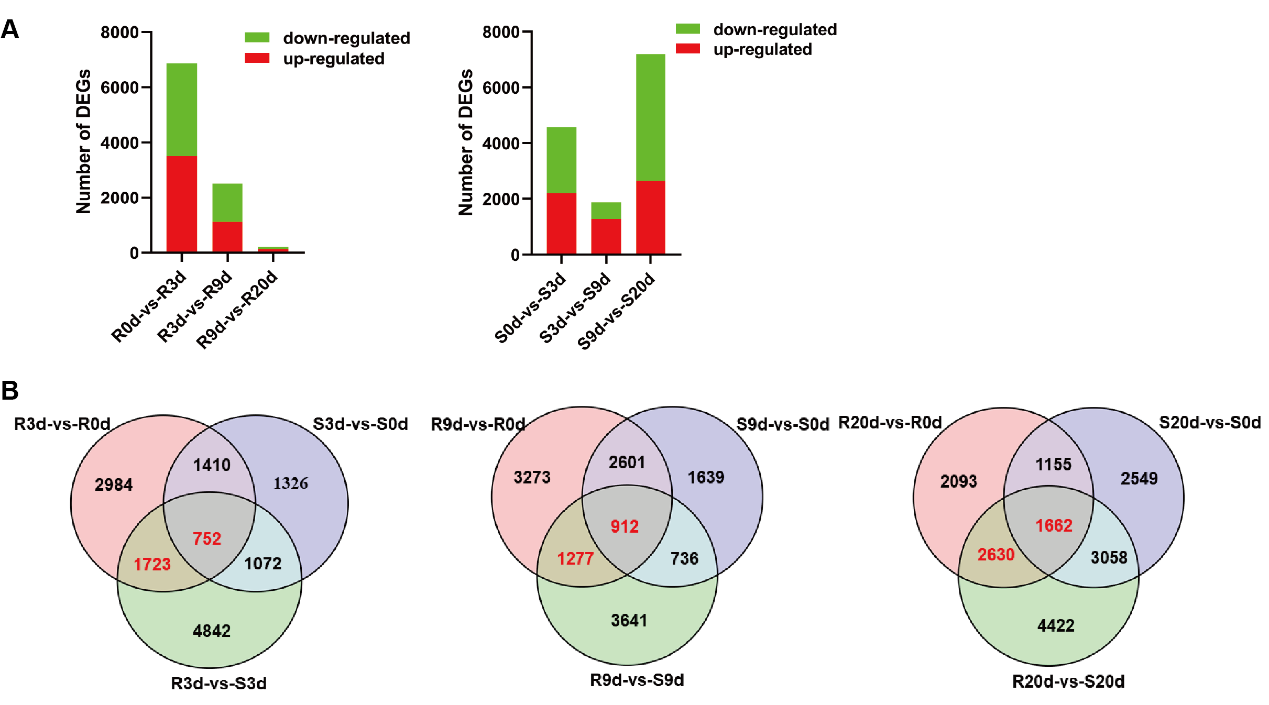


**Figure S3.** Relative DEGs in response to *P. brassicae* in R-line and S-line at different time points.


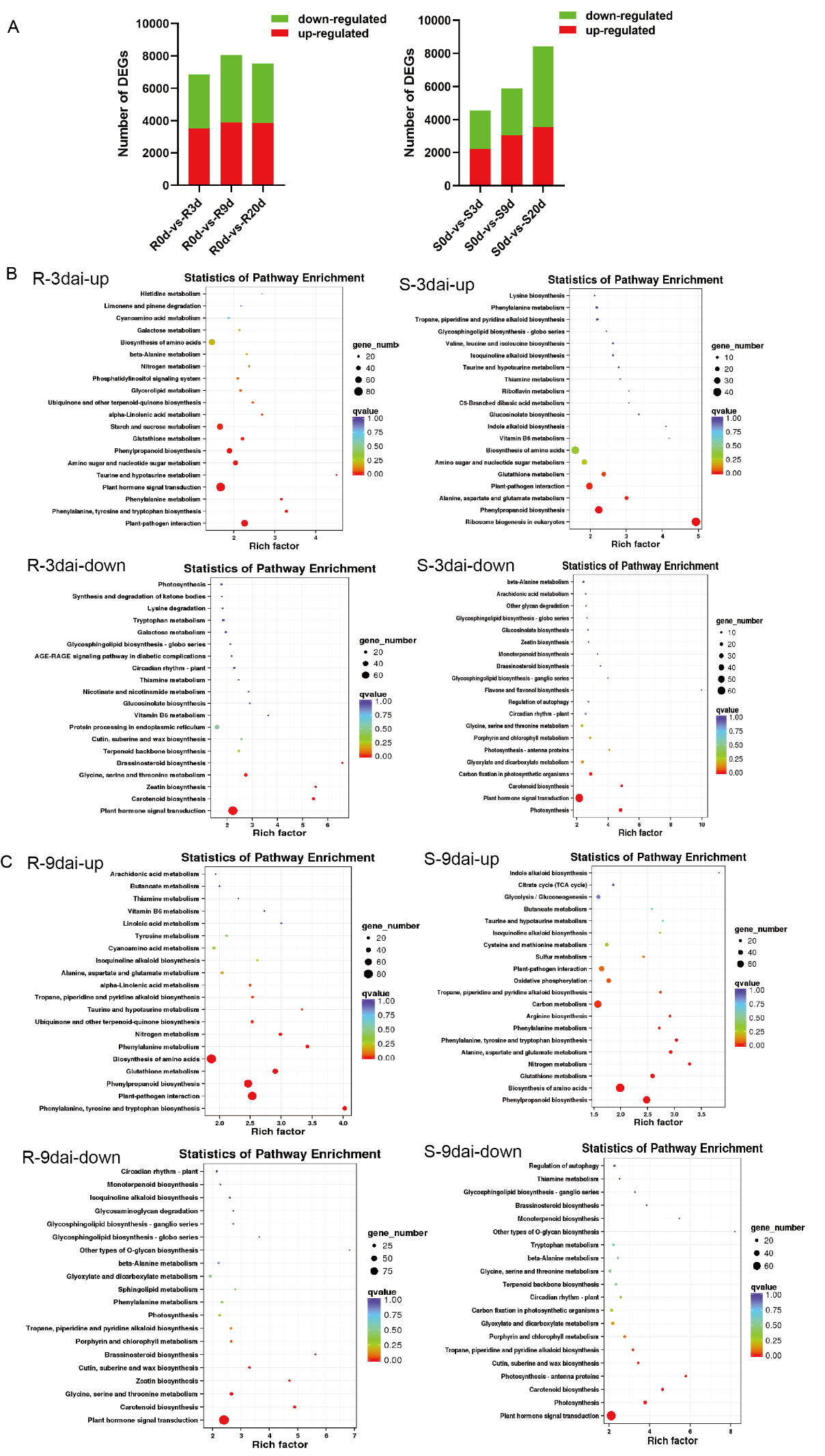


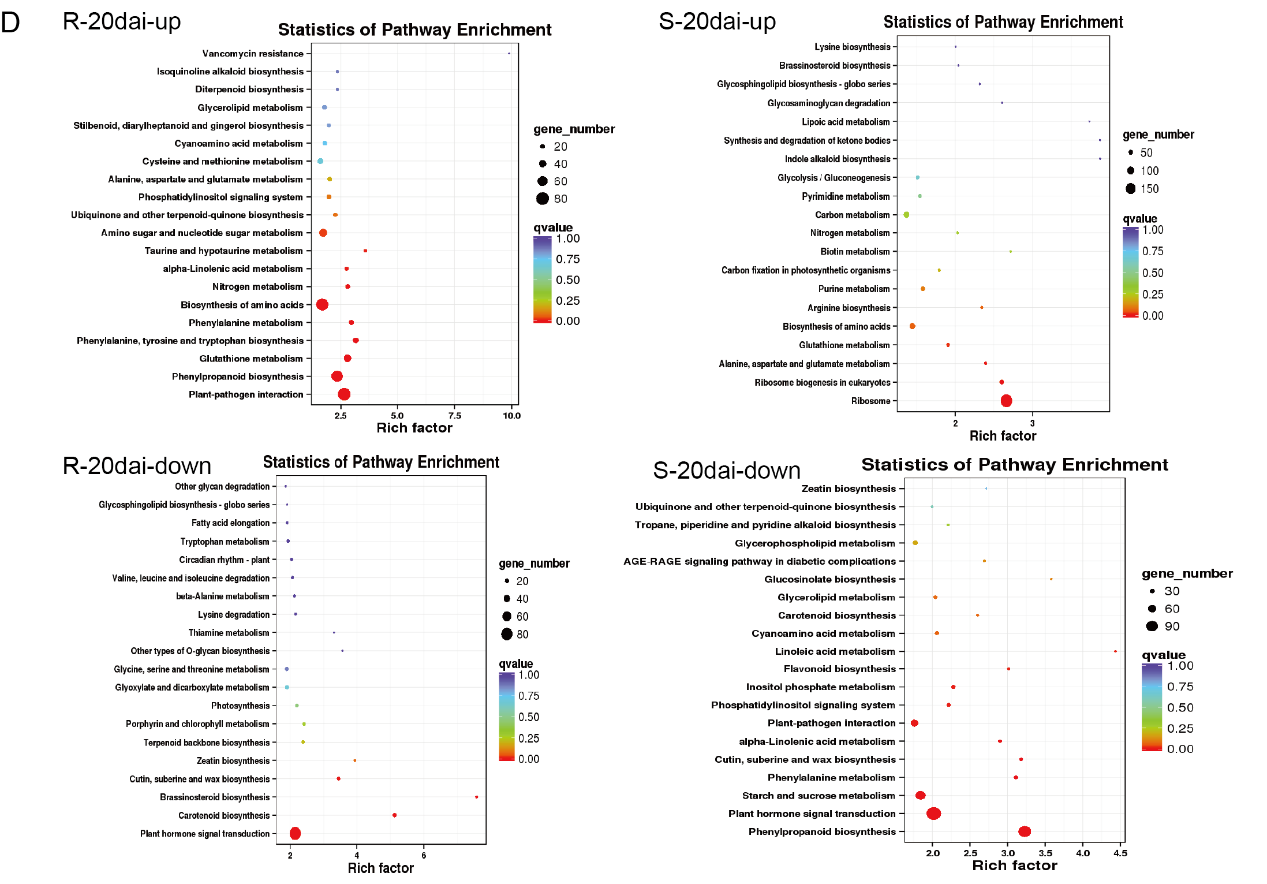


**Figure S4.** KEGG Pathway enrichment analysis of differentially expressed genes between R and S lines at 3, 9 and 20 DAI.

= = ( ) = =
